# Neonatal acute liver failure with pulmonary yellow hyaline membrane and kernicterus

**DOI:** 10.4322/acr.2021.268

**Published:** 2021-05-06

**Authors:** Kei Shing Oh, Hisham F. Bahmad, Carole Brathwaite, Amilcar Castellano Sanchez, Monica Recine

**Affiliations:** 1 Mount Sinai Medical Center, Arkadi M. Rywlin M.D. Department of Pathology and Laboratory Medicine, Miami, FL, USA; 2 Nicklaus Children’s Hospital, Miami, FL, USA; 3 Florida International University, Herbert Wertheim College of Medicine, Miami, FL, USA

**Keywords:** Bilirubin, Kernicterus, Brain, Lung

## Abstract

**Background:**

Neonatal acute liver failure (NALF) is a rare and life-threatening condition. It causes bilirubin to accumulate to a dangerous level in the body, causing permanent damage to vital organs such as the brain and lungs. In many cases, the etiology of NALF remains unknown.

**Case presentation:**

We described a case of an 8-day-old baby girl who presented with poor oral intake, lethargy, and jaundice. Her clinical condition rapidly deteriorated with progression to multi-organ failure, and despite intensive resuscitation efforts, she expired. At autopsy, the most significant findings were liver necrosis, yellow hyaline membrane deposition in the lungs, and bilirubin deposition in the brain (kernicterus).

**Conclusions:**

NALF is a rare and potentially fatal condition necessitating prompt recognition and disease-specific treatment approaches. Toxic accumulation of bilirubin in the lungs can lead to hypoxia and precipitate further ischemic injury to the liver.

## INTRODUCTION

Neonatal acute liver failure (NALF) is a rare and life-threatening condition.^1^ It is defined as a multi-systemic syndrome with liver failure and does not require hepatic encephalopathy as a defining feature. Over the last decade, with changing diagnostic criteria, the treatment approach and outcome have significantly evolved. Although these four grouped etiologies can account for most cases of NALF: Gestational alloimmune liver disease (GALD), viral infections, Hemophagocytic lymphohistiocytosis (HLH), and mitochondrial hepatopathies; in many cases of NALF the etiology is considered “indeterminate.”^1^^,^^2^ NALF causes significant bilirubinemia, which in turn accumulates to dangerous levels and may cause permanent damage to vital organs such as the brain and lungs. Mortality rates for NALF are still as high as 24%.^1^ We discuss the autopsy findings in an 8-day-old girl who presented with lethargy, poor oral intake, and jaundice with rapid progression to multi-organ failure, and review the causes of NALF.

## CASE REPORT

An 8-day-old baby girl born at 37 weeks of gestation to a previously healthy mother via spontaneous vaginal delivery. There were no complications during pregnancy or birth. The patient was brought to the hospital with a 2-day history of lethargy, poor oral intake and jaundice. Physical examination showed that the patient was in respiratory distress. In turn, she was placed on continuous positive airway pressure (CPAP) and admitted to the neonatal intensive care unit (NICU). Laboratory findings on admission revealed significant hyperbilirubinemia with a total bilirubin of 31.5 mg/dL (RV: 0.2-1.3 mg/dL) and direct bilirubin of 2.4 mg/dL (RV: 0.0-0.6 mg/dL). The patient’s condition rapidly deteriorated over 24 hours, with worsening lactic acidosis and coagulopathy. Electroencephalogram (EEG) confirmed seizure activity. An abdominal x-ray showed free air in the abdomen, suggesting abdominal compartment syndrome. Henceforth, an exploratory laparotomy was done; however, no evidence of bowel ischemia or perforation was noted. The decision was made to leave the abdomen open and to place the intestines in a silo bag to assist in resuscitation. Work-up for possible sepsis was pursued, and empiric treatment with broad-spectrum antibiotics and antiviral therapy was initiated. However, the patient’s condition continued to deteriorate with refractory hyperkalemia (>8 mEq/L), cardioplegia, and cardiogenic shock. Due to worsening multi-organ failure, the patient was transferred to a tertiary care center on the same day for further management.

On arrival to the tertiary care center, the patient developed profound hypotension and was placed on three vasopressors. Repeat EEG confirmed seizure activity and severe encephalopathy. Ultrasound of the brain showed increased echogenicity of the bilateral basal ganglia. Laboratory findings revealed prolonged prothrombin time (PT) of 70.8 seconds (RV: 11-13.5 seconds), and prolonged partial thromboplastin time (PTT) of 117.1 seconds (RV: 30-40 seconds), international normalized ratio (INR) of 8.26 (RV: ≤ 1.1), serum ferritin > 21,500 ng/mL (RV: 20-200 ng/mL), lactate dehydrogenase (LDH) of 19,265 IU/L (RV: 580-2000 IU/L), total bilirubin of 7.5 mg/dL (RV: 0.2-1.3 mg/dL), conjugated bilirubin of 3.3 mg/dL (RV: 0.0-0.6 mg/dL), unconjugated bilirubin of 3.9 mg/dL (Reference: 0.5-10.5 mg/dL), alanine transaminase of 210 IU/L (RV: 8-32 IU/L), aspartate aminotransferase of 1,873 IU/L (RV: 24-72 IU/L), and alkaline phosphatase of 270 IU/L (RV: 65-365 IU/L).

Resuscitation attempts for several hours including emergent extracorporeal cardiopulmonary resuscitation (ECPR)/extracorporeal membrane oxygenation (ECMO) without any response and eventually she expired a few hours later.

## AUTOPSY PRESENTATION

**Figure 1:**
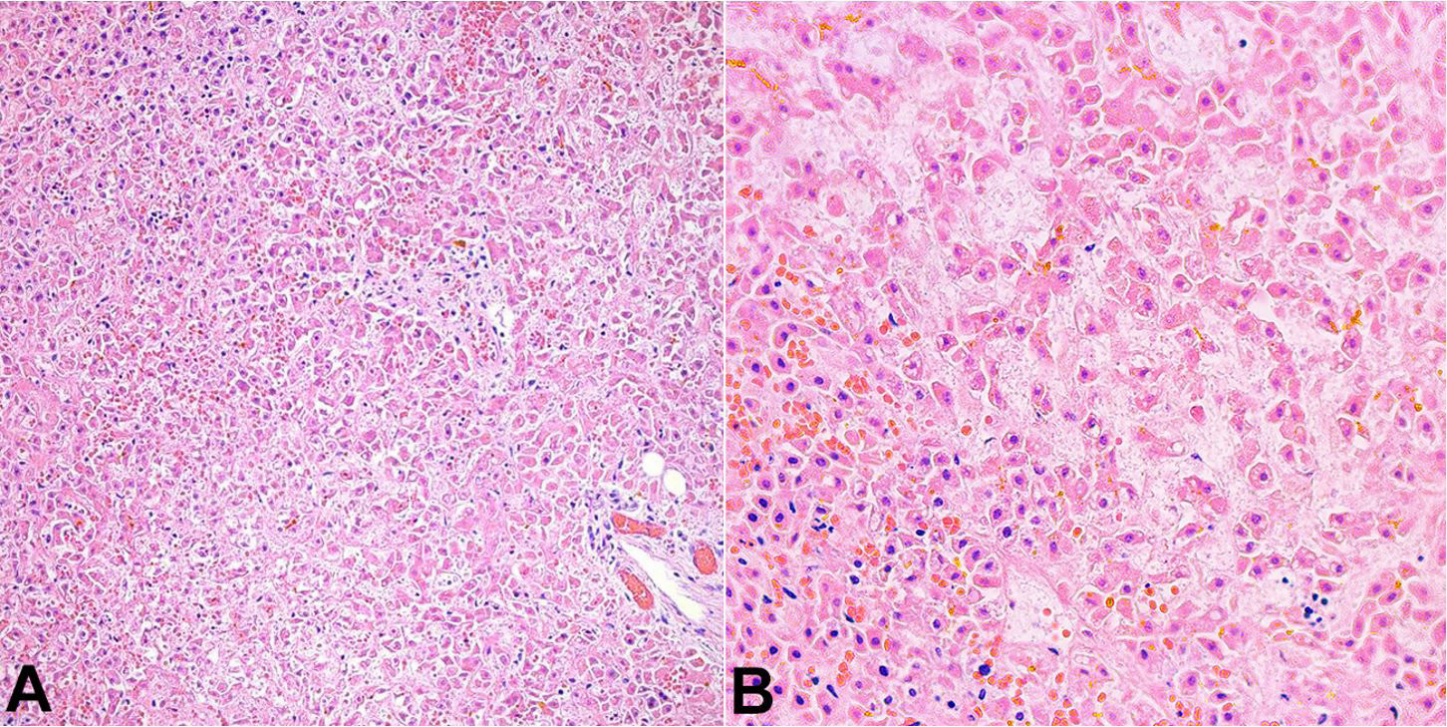
Microscopic image of liver showing patchy areas of necrosis (H&E; 20x in **A** and 40x in **B)**.

**Figure 2:**
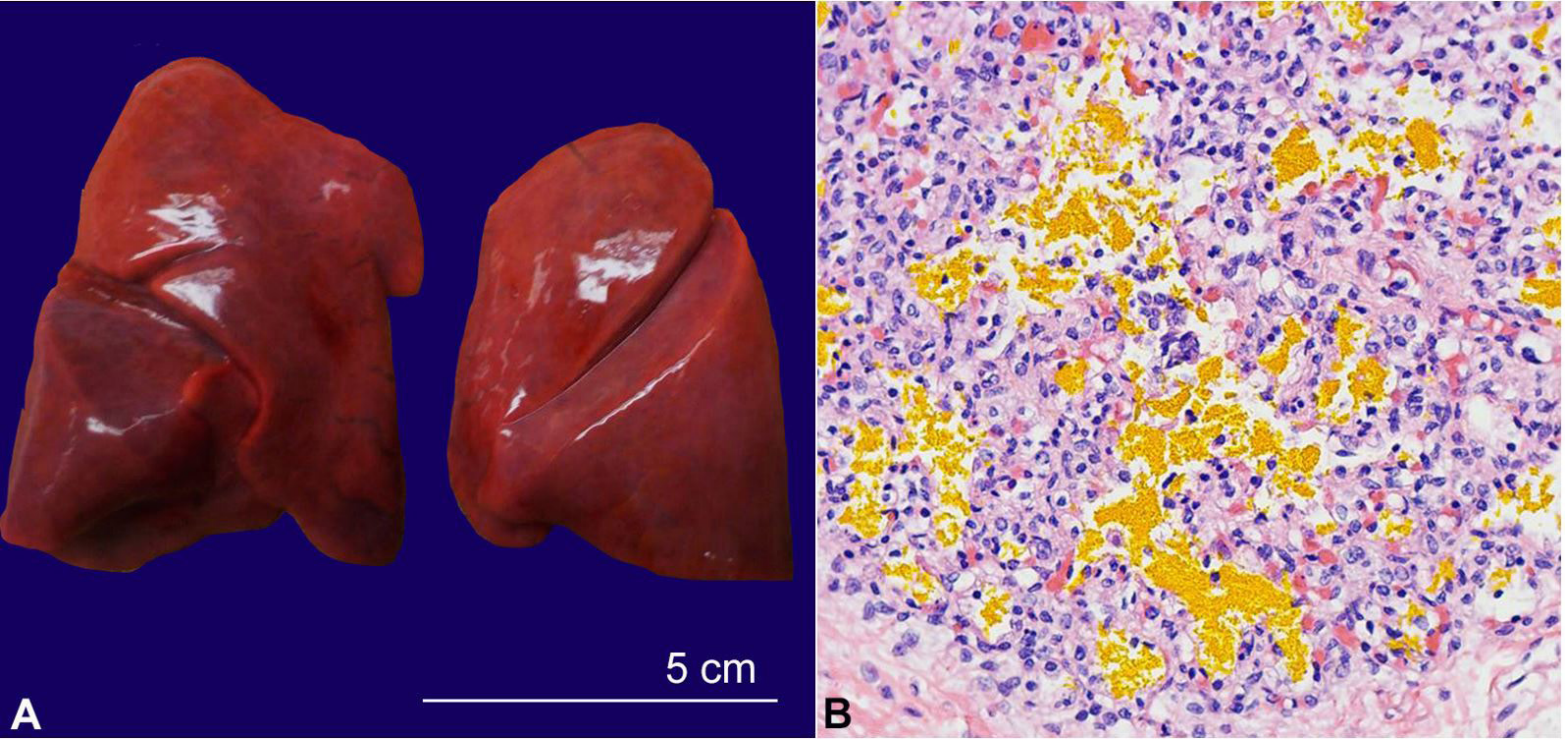
Yellow hyaline membrane disease. **A –** Gross image showing bilateral congested lungs with slight yellow discoloration; **B –** Microscopic image showing extensive intra-alveolar bilirubin pigment deposition. (H&E, 40x).

At autopsy, the pertinent significant findings included: yellow discoloration of the liver, predominantly noted on the right lobe. Microscopic examination showed hepatic congestion and geographical ischemic necrosis along with intracytoplasmic, canalicular and ductular cholestasis (Figure 1). The major bile ducts and gallbladder were unremarkable. Prussian stain failed to demonstrate any extrahepatic iron deposition. Both lungs showed slight yellow discoloration and weighed appropriate to age (right: 40 grams, left: 28 grams) (Figure 2A). Microscopic examination showed congestion with extensive deposition of bright yellow pigment within the alveolar spaces (Figure 2B). A portion of the jejunum and ileum identified within a silo bag showed intramural hemorrhage, vascular congestion, and focal necrosis consistent with bowel infarct. The spleen showed congestion. The bone marrow showed trilineage hematopoiesis and appropriate cellularity to age. Both kidneys showed medullary congestion, necrosis and sloughing of the tubular epithelial cells consistent with acute tubular necrosis. Post-mortem blood and lung cultures were consistent with post-mortem contamination.

**Figure 3:**
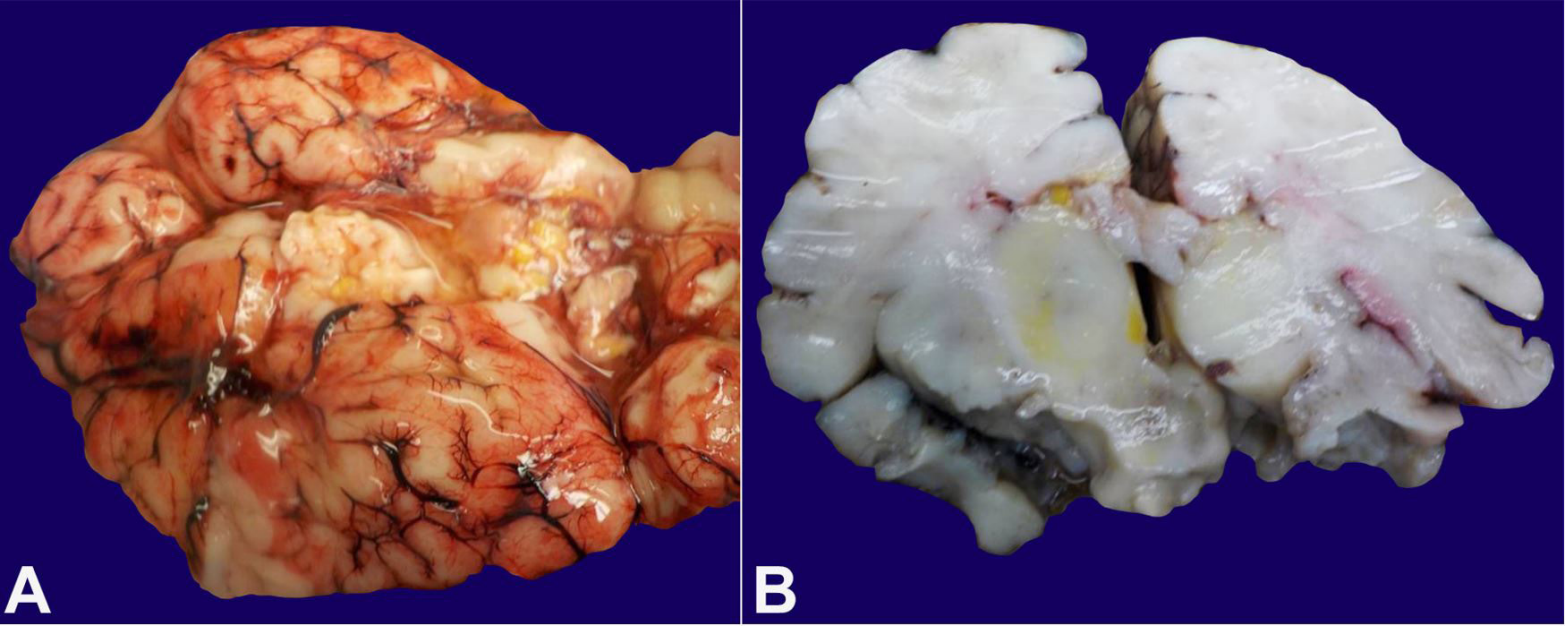
Kernicterus. **A –** Gross image of the brain with bilirubin pigment deposition in the fresh state; **B –** Gross image of coronal section of the brain with bilirubin pigment deposition in the basal ganglia, post-fixation.

**Figure 4:**
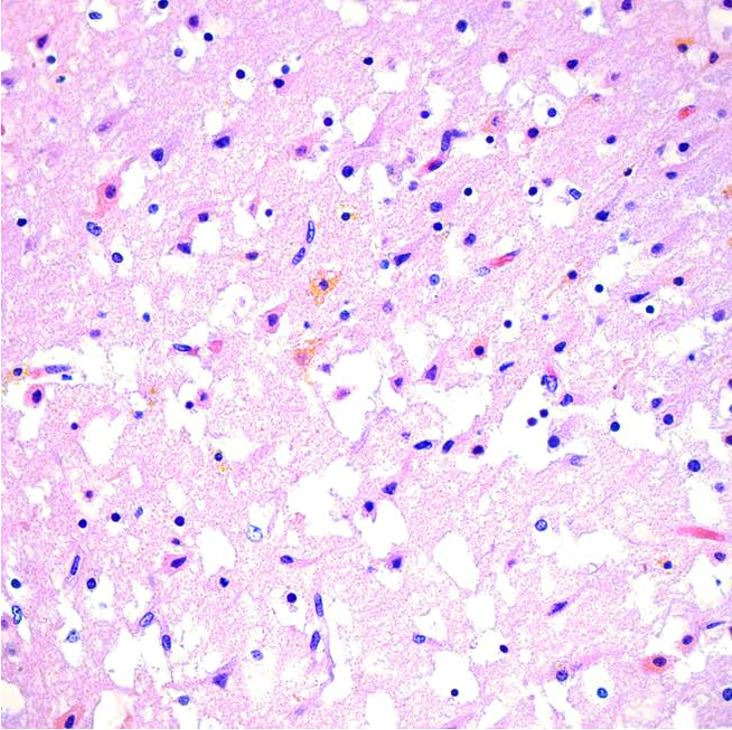
Microscopic image of the cerebral hemisphere showing extensive vacuolation, gliosis, numerous neurons with cytoplasmic eosinophilia and nuclear pyknosis, associated with yellow-orange cytoplasmic pigment deposition (H&E, 40x).

The brain was partially liquefied with extensive bright yellow bilirubin pigment deposition noted in the fresh state (Figure 3A). These pigments were noted most prominently in the basal ganglia and thalamus (Figure 3B) in addition to scattered portions of the brain, including the cerebellum, bilateral occipital lobes, and areas around the lumen of the fourth ventricle. Microscopic examination demonstrated marked distortion of the architecture of the cerebral hemispheres with vacuolation, severe gliosis and numerous neurons with cytoplasmic eosinophilia and nuclear pyknosis, associated with the presence of yellow-orange cytoplasmic pigment (Figure 4). The most extensive damage was noted in the frontal and occipital cortices where there was abundant yellow pigment microscopically, diffuse vacuolation associated with neurons with cytoplasmic eosinophilia and nuclear pyknosis as well as vascular congestion and architectural distortion of the gray and white matter. All these findings were consistent with bilirubin encephalopathy / kernicterus. Gross and microscopic examination of other organs were unremarkable. Liver polymerase chain reaction (PCR) for bacteria, fungus, toxoplasma, adenovirus, parvovirus B19, cytomegalovirus, varicella zoster virus, herpes simplex virus 1 and 2 was negative. Hemophagocytic lymphohistiocytosis (HLH) genetic panel was negative.

## DISCUSSION

Neonatal acute liver failure (NALF) is a rare and poorly understood disease. In adults, acute liver failure (ALF) is defined as an international normalized ratio (INR) of ≥ 1.5 not corrected with parenteral vitamin K, in the presence of hepatic encephalopathy occurring within eight weeks of the onset of jaundice without pre-existing liver disease.^3^ However, because encephalopathy is difficult to assess or quantify in neonates and young children, the Pediatric Acute Liver Failure Study Group (PALF) defines it as: an INR ≥2 without correction with vitamin K, with or without encephalopathy in children aged less than 18 years old.^3^ Neonates with ALF show high mortality rates approaching as high as 24%.^2^ The most common mechanism leading to ALF among this population is acute hepatic necrosis; however, while there are many known causes for NALF, in many cases the etiology remains indeterminate.^2^ Taylor and Whitington^2^ proposed a simplified differential diagnosis for NALF and divided them into four etiologic categories: (i) GALD-NH (Gestational alloimmune liver disease - neonatal hemochromatosis), (ii) viral infections, (iii) HLH, and (iv) mitochondrial hepatopathy. In their experience, GALD was the most common cause of NALF, which is due to complement-mediated hepatocyte injury due to the transfer of maternal IgG across the placenta.^2^ GALD is also the most common cause of neonatal hemochromatosis (NH), characterized by increased iron deposition in extrahepatic tissues such as the salivary glands, thyroid, myocardium, and pancreas.^4^ Neonates with GALD/NH often present with a history of intrauterine growth restriction and signs of liver failure, including bleeding, anasarca, and culture negative sepsis.^2^

Viral infections are also considered a common cause of NALF, herpes simplex virus (HSV) being the most common.^2^ HSV is acquired by the passage through the birth canal of infected mother.^2^ Rarely, other viruses have also been associated with NALF including Human Herpesvirus 6 (HHV6), cytomegalovirus (CMV), enterovirus and adenovirus.^2^ Viral infections can be detected using polymerase chain reaction (PCR) among other methods; PCR was negative in our case.

HLH is another underrepresented cause of NALF.^5^ The disease in neonates and infants can be primary due to a genetic mutation affecting cytotoxic T-cell and Natural Killer-cell function or secondary (due to viral infection or neoplasms), resulting in excessive immune activation;^6^ other etiologies of HLH include viral infections and neoplasms. Diagnostic criteria for HLH include a combination of the following: fever, splenomegaly, pancytopenia, hyperferritinemia (>20,000 ng/mL), hypertriglyceridemia, decreased fibrinogen level, and evidence of hemophagocytosis in the bone marrow, spleen, liver, or lymph node.^7^ Genetic testing for HLH in our case was negative.

Other causes of NALF that do not show hepatic necrosis include mitochondrial and metabolic disorders such as: tyrosinemia, galactosemia, and hereditary fructose intolerance.^1^ Hypoxic-ischemic injury is sometimes associated with NALF; however, the newborn liver is relatively refractory to hypoxic insult.^2^^,^^6^ Furthermore, other signs of circulatory failure leading to hypoxia/tissue ischemia usually precede signs of liver injury. Our case disclosed hypoxic ischemic damage to the central nervous system.

As a result of acute liver injury, bilirubin level increases beyond the capacity of which the newborn may process, leading to accumulation and widespread deposition in vital organs. Kernicterus is traditionally used to describe the pathological yellow staining (icterus) of the deep nuclei (“kernel”) of the brain. It is a permanent disabling condition usually encountered when serum unconjugated bilirubin level exceeds 25 mg/dL [8]. This condition is characterized by extrapyramidal movement disorders such as dystonia and choreoathetosis, hearing loss, and oculomotor paresis, reflecting the regional central nervous system (CNS) topography of bilirubin-induced damage.^8^

Another prominent finding in our case was the extensive yellow pigment deposition in the lungs. The deposition of bright yellow pigment in the lungs, termed “yellow hyaline membrane disease” (YHMD), was first described at the beginning of 1965.^9^ This observation coincides with the initiation of an active intensive care unit for premature infants and prolonged survival of severe respiratory distress syndrome cases.^9^ In one of the most extensive morphological study on YHMD published by Turkel and Mapp^10^ in 1983, where a total of 667 cases with hyaline membrane disease (499 with pink, and 168 with yellow membranes) were included, it was reported that YHMD was a more frequent finding among premature infants who survived longer. Grossly, YHMD is characterized by yellow coloration that does not fade on exposure to air, light, or prolonged refrigeration.^11^ Microscopically, the yellow hyaline membranes (YHMs) are visible as a permanent bright yellow pigment which are unaffected by conventional hematoxylin and eosin staining. The yellow pigment can be seen lining the alveolar space, diffusely within hyaline membranes, or freely within the alveolar and bronchiolar lumen, or macrophages.^11^ Valdés-Dapena et al.^9^ used Hall’s stain to identify the presence of yellow bilirubin pigment in YHMD. By electron microscopy, the yellow membranes consist of needle-like structures varying in length from 2.500 Ǻ to 5.000 Ǻ and in width from 250 Ǻ to 400 Ǻ, interpreted as either bilirubin-protein or bilirubin-lipoprotein complexes.^9^ Using scanning spectrophotometer, Morgenstern et al.^12^ identified that the homogenates of lungs with YHMs showed a unique absorption shoulder at 454 nm, which corresponded to the absorbance of unconjugated bilirubin. Additionally, thin layer chromatography of the extracted yellow hyaline membrane material produced two unique spots similar to those of chemically pure bilirubin.^12^ Interestingly, unlike bilirubin deposition in other body sites causing icterus and kernicterus, the presence of the YHMs does not correlate with the increased peak level of serum bilirubin.^11^ It was believed that mild hyperbilirubinemia, in the presence of other factors such as hypoproteinemia, and decreased bilirubin binding to albumin could result in diffusion through alveolar walls damaged by prolonged shock and the deposition of unconjugated bilirubin on the previously formed intra-alveolar hyaline membranes.^10^ Another study also suggests the role of acute pulmonary hemorrhages in the formation of yellow hyaline pigment in the lungs.^13^

## CONCLUSION

In summary, we reported a case of NALF, presenting with rapidly progressive multi-organ failure. Significant autopsy findings included liver and kidney damage, kernicterus and prominent yellow hyaline membrane disease. Although two of the leading causes of NALF were ruled-out in this case (HLH and viral infection), the cause of acute liver failure remains indeterminate as the possibility of alloimmune associated hepatitis and other rare mitochondrial/metabolic disorders could not be ruled out.
